# Closed total talus dislocation without fracture: a case report

**DOI:** 10.1186/1757-1626-2-9132

**Published:** 2009-12-02

**Authors:** Seyed Reza Sharifi, Mohamad H Ebrahimzadeh, Hosein Ahmadzadeh-Chabok, Javad Khajeh-Mozaffari

**Affiliations:** 1 Orthopedic Research Center, Mashad University of Medical Sciences, Mashad 91766-99199, Iran

## Abstract

Total dislocation of the talus from all of its joints is a rare injury specially when the talus and malleoli are not fractured and frequently it is as a result of a high-energy trauma. It usually leads to degenerative changes in neighboring joints and frequently avascular necrosis is a predictable outcome. We present a case of total talus dislocation because of a high-energy trauma in association with other major fractures resulting from a fall from height, but no fracture could be detected in the talus and any of malleols. Closed reduction was unsuccessful and we performed open reduction. At 6 month post operation follow-up, the talus didn't show subluxation and avascular necrosis could not be detected.

## Introduction

Total dislocation of the talus bone from all of its three joints (tibiotalar, subtalar, and talonavicular) is a very rare event. It is caused by a high energy trauma and indicates a sever injury with disruption of almost all ligaments and capsular attachments of the talus.

Total dislocation of the talus commonly is associated with fractures, either malleolar fracture or talus fracture[1,2]. Closed dislocations without concomitant fracture of malleoli and the talus itself, occurs even more scantly and when happened, usually are open injuries[3,4]. Closed total dislocation of the talus is a case worthy to be reported, as the literature indicates so. In this case report, we present a patient who sustained a high-energy trauma because of a fall from height with total dislocation of the talus, without malleolar or talus fracture. Its management, procedures undertaken and postoperative care are described.

## Case report

A 28-years-old young man who received a sever impact on his left lower limb because of falling (40 feet height) was brought to the emergency department of our referral level 1 trauma center. On admitting, he was hemodynamically stable. Mild respiratory distress was present. Skin laceration in left frontal region of skull and a perioccular eccymosis on his left side could be seen. He was clearly alert (GCS:15) and he later proved to have no neurosurgical injury.

His entire left lower limb was externally rotated and the injured foot appeared swollen, tense and deformed with a medially shifted hind foot and a supinated fore foot. Tibialis posterior and dorsalis pedis pulses of the extremity were normal. His left elbow was deformed with a 2 cm open wound; and deformation and swelling could be seen in his left wrist. Radial pulse was weak with delayed capillary filling.

**Figure 1:**
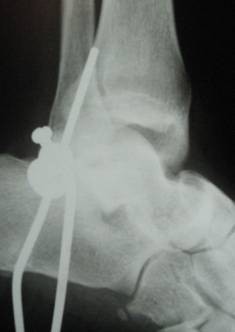
**lateral radiograph of the ankle showed total lateral talus dislocation**.

**Figure 2:**
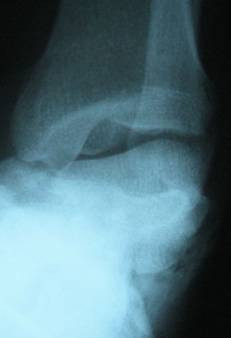
**Antero-posterior radiograph of the ankle showed total lateral talusr dislocation**.

**Figure 3:**
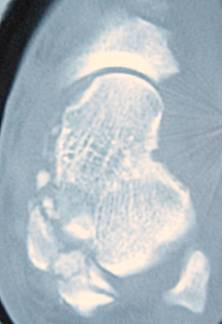
**CT scan of the ankle**.

After a comprehensive clinical evaluation, essential plain radiographies were taken in emergency department. Radiographies of extremities presented posterolateral left elbow dislocation, comminuted left distal radius fracture, comminuted left intertrochanteric and subtrochanteric fracture and a closed total dislocation of the talus with concomitant cuboid fracture (Fig 1, 2,3).

Chest x-ray showed interstitial lung contusion, pneumothorax and hemothorax. In plain radiography of the pelvis, a transverse fracture of left pubic ramus could be detected while CT Scan later revealed ipsilateral stable sacral fracture.

Closed manipulation of talus dislocation was tried with the aid of a calcaneal pin but C-arm proved the reduction to be unsuccessful. We attempted open reduction via an anteromedial approach.

**Figure 4:**
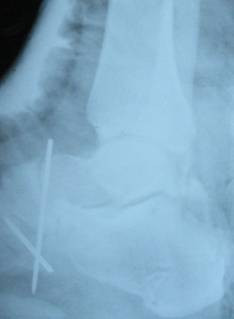
**Post closed reduction X-ray**.

Irreducible subtalar joint was responsible for unsuccessful closed reduction. The head of talus was trapped between flexor hallucis longus and flexor digitrum longus tendons. All ruptured elements was repaired. We checked the neurovascular status of the foot which was normal and then we applied a short leg cast (Fig 4).

Concentrating on his left foot; he was prescribed a protected weight bearing with only toe touch at the beginning. Post-operative radiographies were taken at weekly intervals for the first four weeks, to rule out subluxation; while complete weight bearing, out of cast was allowed at 3 months post injury after removing cast, the patient started an observed physiotherapy for 2 months. X-Ray of the ankle didn't show Hawkin's sign but AVN could not be detected as well. We followed-up the patient untile 1 year post-operativly and we did not see AVN of the talus or post-thatic arthritis.

## Discussion

Total dislocation of talus bone from all of its three joints (tibiotalar, subtalar, talonovicular) is an unusual injury that results from high-energy trauma. This type of injury is supposed to be in continuation of subtalar dislocation when the force magnifies and continues. Dislocation of subtalar joint is the first stage of the injury. When the force progress, talonavicular joint dislocates and finally tibiotalar joint dislocation occurs. The mechanism is thought to be excessive supination or pronation. Supination force results in lateral dislocation and pronation force lead in medial dislocation. In closed reduction, trying to reduce tibiotalar joint is the key for successful treatment. With reduction of tibiotalar joint, other joints (subtalar and talonavicular) spontaneously reduce. [5,6]

In the present case the mechanism of injury is axial loading because of falling with or without supination or pronation injury. Because of high energy nature of trauma, combination of mechanisms are responsible and an exact isolated mechanism can not be clarified. Close reduction was not successful and the main reason appeared to be subtalar joint locked in dislocated position and trapped talar neck between flexor tendons. Open reduction via an anteromedial approach with dislodgment of subtalar join, easily reduced the dislocation and all other dislocations subsequently reduced.

Because all capsular and ligamentous attachments of the talus ruptures in this injury and dependence of talus bone vascularization on this elements, avascular necrosis of the talus is mostly predictable. Although some investigators believe that avascular necrosis is inevitable consequence of the injury, there are some reported cases of total talar dislocations without osteonecrosis. Reduced range of motion and osteoarthritis in any three joint are also predictable complications. [6-8]

## Conclusion

Total dislocation of the talus is a rare injury, usually as a result of a fall from hieght trauma. Usually total talus dislocation accompanies other fractures of neighboring bones as malleolar fracture or talus fracture itself and usually it is an open injury. But we report a closed total talus dislocation without malleolar or talar fractures. It required open reduction after an unsuccessful closed reduction attempt. The main obstacle to closed reduction appeared to be the trapped talar neck between flexor tendons and irreducible subtalar joint. This injury frequently leads to degenerative changes in related joints, decreased range of motion of the ankle and avascular necrosis of the talar body. When avascular necrosis does not develop, it is maybe because of retained blood supply through deltoid branch or posterior process branch, but it remains to be investigated further.

## Consent

The patient has signed out a formal consent form for publication of this case report and pictures.

## Competing interests

The authors declare that they have no competing interests.

## Authors' contributions

SSR, KMM, MHE admitted the patient and performed surgery, MHE, KMM, ACH followed the patient and contributed in writing the MS.
